# Biosynthesis of zinc oxide nanoparticles by cell-biomass and supernatant of *Lactobacillus plantarum* TA4 and its antibacterial and biocompatibility properties

**DOI:** 10.1038/s41598-020-76402-w

**Published:** 2020-11-17

**Authors:** Hidayat Mohd Yusof, Nor’Aini Abdul Rahman, Rosfarizan Mohamad, Uswatun Hasanah Zaidan, Anjas Asmara Samsudin

**Affiliations:** 1 Department of Bioprocess Technology, Faculty of Biotechnology and Biomolecular Sciences, Universiti Putra Malaysia, 43400 Serdang, Selangor Malaysia; 2 Bioprocessing and Biomanufacturing Research Centre, Faculty of Biotechnology and Biomolecular Sciences, Universiti Putra Malaysia, 43400 Serdang, Selangor Malaysia; 3 Department of Biochemistry, Faculty of Biotechnology and Biomolecular Sciences, Universiti Putra Malaysia, 43400 Serdang, Selangor Malaysia; 4 Department of Animal Science, Faculty of Agriculture, Universiti Putra Malaysia, 43400 Serdang, Selangor Malaysia

**Keywords:** Biological techniques, Biophysics, Biotechnology, Microbiology, Systems biology, Materials science

## Abstract

This study aims to utilize the cell-biomass (CB) and supernatant (CFS) of zinc-tolerant *Lactobacillus plantarum* TA4 as a prospective nanofactory to synthesize ZnO NPs. The surface plasmon resonance for the biosynthesized ZnO NPs-CFS and ZnO NPs-CB was 349 nm and 351 nm, respectively, thereby confirming the formation of ZnO NPs. The FTIR analysis revealed the presence of proteins, carboxyl, and hydroxyl groups on the surfaces of both the biosynthesized ZnO NPs that act as reducing and stabilizing agents. The DLS analysis revealed that the poly-dispersity indexes was less than 0.4 for both ZnO NPs. In addition, the HR-TEM micrographs of the biosynthesized ZnO NPs revealed a flower-like pattern for ZnO NPs-CFS and an irregular shape for ZnO NPs-CB with particles size of 291.1 and 191.8 nm, respectively. In this study, the biosynthesized ZnO NPs exhibited antibacterial activity against pathogenic bacteria in a concentration-dependent manner and showed biocompatibility with the Vero cell line at specific concentrations. Overall, CFS and CB of *L. plantarum* TA4 can potentially be used as a nanofactory for the biological synthesis of ZnO NPs.

## Introduction

Zinc oxide nanoparticles (ZnO NPs) possess unique characteristics that include chemical, physical, optical, and biological properties. These nanoparticles (NPs) have been widely applied in many industries such as environmental, catalyst, optical, agriculture, and biomedical^1,2^. ZnO NPs are mainly used as antimicrobial agents in wound dressings, textiles, and food packaging^3,4^. Thus, the efficacy of ZnO NPs in in vitro antimicrobial activity has been tested on a wide range of pathogenic microorganisms^3,5^. In general, the antimicrobial activity of ZnO NPs depends on their size and shape. The high surface to volume ratio of ZnO NPs increases their reactivity, which then amplifies their antimicrobial effects^6^. This is also attributed to the differences in properties between ZnO NPs and their bulkier counterparts. Currently, researchers are focusing on the development of NPs as an alternative to antibiotics due to the increase in multidrug-resistant bacteria. On the other hand, ZnO NPs are also used as a feed supplement in animal diet^7–11^. Zinc is an essential trace element that plays a very important role in the body’s biological function^12^. Moreover, zinc in the form of NPs will increase its absorption and bioavailability in the body.

Although ZnO NPs have many advantageous applications, they also present toxic effects on human and animal cells. Several in vitro studies have demonstrated the potential toxic effects of ZnO NPs on various cell lines^13,14^. These cytotoxic effects include the induction of oxidative stress and cellular damage^15^ associated with the release of free Zn^2+^ ions from ZnO NPs, which subsequently cause cell damage. Nevertheless, the report of ZnO NPs’ cytotoxicity effects on cell lines is inconsistent. Previous studies have suggested that the cytotoxicity effects of NPs are influenced by their size, shape, and dosage^12,16,17^. Moreover, the method used for the synthesis process has been reported to contribute to the toxicity effects of NPs due to the chemical reaction conditions in the chemical method that can potentially limit NPs’ biological applications^18^. Therefore, the development of ZnO NPs with high activity and low toxicity is needed.

Conventionally, high purity ZnO NPs can be effectively produced on a large scale through chemical and physical methods. Despite the advantages of these methods, there are major drawbacks concerning their high production cost and environmental impact due to the use of harsh chemicals in the synthesis processes that generate hazardous wastes and contribute to the innate toxicity of NPs. Consequently, the interest in developing a more environmentally benign, biocompatible, simple, and inexpensive synthesis method of ZnO NPs has been increasing. For example, the use of microorganisms such as bacteria, fungi, and yeast to synthesize ZnO NPs is considered a great alternative to conventional chemical and physical synthesis methods^2^. Biological synthesis of NPs using microorganisms seems to be a more environmentally sustainable NPs production and has drawn much interest compared to plants because microorganisms are easily cultured without seasonal and geographical area restrictions. Moreover, using bacteria for the biosynthesis of ZnO NPs has significant advantages due to the production of functional biomolecules in the supernatant, which can reduce the metal ions into metal NPs^12,19^. In addition, the cell biomass of bacteria could act as a nanofactory in ZnO NPs’ production due to the presence of a functional group on the bacterial cell that reduces the metal ions into metal NPs^20,21^.

Among the bacteria used for ZnO NPs synthesis, probiotic lactic acid bacteria (LAB) has received great interest due to its non-pathogenic and beneficial properties. LAB is a Gram-positive bacteria with a thick cell wall consisting of numerous biostructures and functional groups^22^. These functional groups act as a ligand for the metal ions to facilitate the formation of ZnO NPs. Furthermore, LAB secretes various enzymes that act as a reducing and stabilizing agent for ZnO NPs. Hence, several studies have been conducted to determine the efficacy of probiotic LAB in mediating the biosynthesis of ZnO NPs using either cell-biomass or cell-free supernatant^23,24^. Nonetheless, to date, the ability to use both routes to mediate the biosynthesis of ZnO NPs remains unexplored.

In our previous study, a zinc-tolerant probiotic, *Lactobacillus plantarum* TA4, which demonstrated its ability in simultaneously resisting high zinc concentration and producing ZnO NPs, has been successfully isolated^25^. Thus, this present study aimed to use cell-biomass (CB) and cell-free supernatant (CFS) derived from *L. plantarum* TA4 as a reducing agent in the biosynthesis of ZnO NPs. UV–Vis spectroscopy, high resolution-transmission electron microscopy (HR-TEM), dynamic light scattering (DLS), and Fourier-transform infrared spectroscopy (FTIR) were used to demonstrate and compare the characteristics of the biosynthesized ZnO NPs. Besides, the antimicrobial activity against Gram-positive and Gram-negative pathogens was determined. Lastly, the biocompatibility of the biosynthesized ZnO NPs, in relation to cytotoxicity in Vero cell line, was also investigated.

## Materials and methods

### Bacterial strain and chemicals

The metal oxide precursor, zinc nitrate, Zn(NO_3_)_2_·6H_2_O, was purchased from Oxoid. The microorganism, *L. plantarum* TA4, which was isolated previously from *tapai pulut* (a local fermented food)^25^, was used to synthesize ZnO NPs and was routinely cultivated in De Man, Rogosa and Sharpe (MRS) broth.

### Biosynthesis of ZnO NPs by *L. plantarum* TA4

For the biosynthesis study, *L. plantarum* TA4 was inoculated in a 250 Erlenmeyer flask containing 100 mL MRS broth and incubated at 37 °C for 24 h with 150 rpm agitation. Upon the completion of incubation, the culture was centrifuged at 2800×*g* for 10 min, and both cell biomass (CB) and cell-free supernatant (CFS) were collected for subsequent study. Briefly, for the CB route, the biomass was washed three times with phosphate-buffered saline (PBS) before suspended into 50 mL of sterilized deionized water containing 500 mM of Zn^2+^ concentration. The suspension was then incubated for 24 h at 37 °C with 150 rpm agitation to initiate the biosynthesis process. Subsequently, the biomass was collected by centrifugation, and the biosynthesized ZnO NPs were acquired by ultrasonic disruption at 30 °C for 30 min. The biosynthesized ZnO NPs were obtained by high-speed centrifugation (18,000×*g* for 30 min), and the collected ZnO NPs were dried at 100 °C. Meanwhile, for the CFS route, the supernatant was used in the process of ZnO NPs synthesis. Briefly, 100 mL of CFS was added to 100 mL deionized water containing 100 mM of Zn^2+^ concentration and then incubated at room temperature overnight with 150 rpm agitation. The appearance of the faded white solution indicated the formation of ZnO NPs. Consequently, the ZnO NPs were collected by centrifugation (18,000×*g* for 10 min) and washed with distilled water and followed by ethanol to remove remaining Zn^2+^ during the reaction mixture before subjected to centrifugation. The collected ZnO NPs were then dried at 100 °C.

### Production of extracellular protein

The Bradford assay study was conducted to examine the possible role of extracellular protein produced by CB and its presence in CFS regarding the formation of ZnO NPs. Briefly, *L. plantarum* TA4 was grown overnight in the MRS medium at 37 °C with 150 rpm agitation. Following incubation, the culture medium was centrifuged at 2800×*g* for 10 min to collect the CB and CFS. The CB was suspended in phosphate buffer saline (PBS) and incubated overnight and centrifuged to obtain the suspension containing protein. The extracellular protein of CB suspension and CFS were determined using the Bradford protein assay (Bio-Rad Laboratories Ltd., UK), according to Liang et al.^26^. Bovine serum albumin was used as the standard. The experiments were conducted in triplicates, and the standard deviation was calculated.

### Characterization of biosynthesized ZnO NPs

To validate the formation of ZnO NPs, the UV–Vis spectroscopy analysis was carried out using Uviline 9400 (Secomam, France) at the wavelength range of 300–700 nm operated at a resolution of 1 nm, and distilled water was used as the blank. Subsequently, the possible biomolecules present in the CB, CFS as well as in the obtained ZnO NPs that were responsible in the reduction, capping or stabilization of NPs processes, were analyzed by FTIR in the range of 400–4000 cm^−1^ at a resolution of 4 cm^−1^ using Nicolet 6700 (Thermo Scientific, US). Briefly, the dry biomass and cell-free supernatant of strain TA4 were collected before the analysis. The obtained dried powder form of biosynthesized ZnO NPs-CFS and ZnO NPs-CB were also analyzed. The hydrodynamic diameter and polydispersity index (PDI) of biosynthesized ZnO NPs in a colloidal form were measured by dynamic light scattering (DLS) using a Nano S (Malvern Instruments, UK). The NPs were dispersed in deionized water. All values were generated and obtained using the software equipped with the instrument. The particle size measurement obtained by DLS was used to compare the size of those obtained from transmission electron microscopy (TEM). High-resolution transmission electron microscopy (HR-TEM) was carried out to characterize the morphology and size of the biosynthesized ZnO NPs. Briefly, the ZnO NPs powder was suspended in ethanol, and a drop of ZnO NPs solution was placed on a copper grid and air-dried. Images were viewed using JEM-2100F (JEOL, Japan). The particles size distribution of biosynthesized ZnO NPs were analyzed using ImageJ software (National Institute of Health, US) with at least 100 particles was measured to obtain the average size.

### Scanning electron microscope (SEM) and energy dispersive X-ray (EDX) analysis of *L. plantarum* TA4 cell biomass exposed to Zn^2+^

To determine the formation of ZnO NPs on the cell biomass of *L. plantarum* TA4 exposed to Zn^2+^, scanning electron microscopy (SEM) equipped with EDX analysis was carried out. Briefly, the cell biomass of *L. plantarum* TA4 was added to the Zn^2+^ aqueous solution at a concentration of 500 mM and incubated for 24 h at 37 °C. After the incubation, the biomass was collected by centrifugation at 2800×*g* for 10 min and were subjected to sample processing for SEM. The SEM observation was performed using JSM-6400 (JEOL, Japan), and the elemental analysis of biomass was conducted using the EDX spectrometer equipped with SEM.

### Evaluation of in vitro antimicrobial activity of biosynthesized ZnO NPs

Antimicrobial activity was measured using the agar well diffusion method against Gram-negative (*Escherichia coli* and *Salmonella* sp.) and Gram-positive (*Staphylococcus aureus* and *Staphylococcus epidermidis*) pathogen. Briefly, microbial strains were cultured until the turbidity of 0.5 of McFarland standard was achieved. Using a sterilized cotton bud, the cultures were swabbed evenly on the nutrient agar plate. Next, the wells were made using a sterile cork-borer with a diameter of 6 mm. About 100 µL of biosynthesized ZnO NPs with a series of concentrations (1000, 2000, 3000, 4000, and 5000 µg/mL) were pipetted into each well and incubated at 37 °C for 24 h. Following incubation, the diameter (mm) of the inhibition zone was measured and recorded. The experiments were carried out in triplicates.

### Determination of minimum inhibitory concentration (MIC) and minimum bactericidal concentration (MBC) of biosynthesized ZnO NPs

The stock solution (10,000 µg/mL) of ZnO NPs were prepared in dH_2_O and sonicated to obtain a homogenous solution. The stock was diluted to obtain a various range of concentrations (10,000–250 µg/mL). The MIC was determined according to the method of Rajeswaran et al.^27^ with some modifications. Briefly, about 0.5 mL of ZnO NPs of each concentration was added to 2 mL of nutrient broth and inoculated with 0.5 mL of bacterial culture in all test tubes. The uninoculated nutrient broth was used as the control, and all the test tubes were incubated for 24 h at 37 °C. After the incubation time, the optical density (OD) was measured at 600 nm in the UV–Vis spectrometer. The concentration of ZnO NPs at which no increase in OD observed was considered as the MIC. Later, the MBC assay was determined by re-culturing each sample from the MIC broth onto nutrient agar plates, and the concentration that showed no growth of bacterial strains was considered as the MBC. All experiments were performed in triplicates.

### Biocompatibility assay

The cell viability study was conducted using in vitro MTT (3-[4, 5-Dimethyl thiazol-2-yl]-2, 5-diphenyltetrazolium bromide) assay on Vero cell line (kidney of African green monkey) to determine the biocompatibility of the ZnO NPs. The Vero cell line was obtained from the UPM-MAKNA Cancer Research Laboratory (CanRes), Bioscience Institute, Universiti Putra Malaysia, Malaysia. In brief, cell culture with a concentration of 2 × 10^3^ cells/mL was prepared and plated (100 µL/well) onto 96-well plates. The cell cultures were then treated with various concentrations of biosynthesized ZnO NPs-CFS and ZnO NPs-CB (15.6, 31.3, 62.5, 125, 250, 500, and 1000 µg/mL) and incubated for 24 h. At the end of the incubation time, MTT reagent was added to the wells and incubated for another 3 h at 37 °C. Further, the observed purple formazone crystals were dissolved with 100 µL of dimethyl sulfoxide (DMSO). The color intensity was measured at 570 nm using an enzyme-linked immunosorbent assay (ELISA) reader. The experiments were performed in triplicates, and the graphs were plotted with the percentage of cell viability against their respective concentrations. The percentage of cell viability was calculated as follows:

$$\%\,cell\,viability= \frac{OD\,of\,treated\,samples}{OD\,of\,untreated\,sample} \times 100.$$

## Results and discussion

### Biosynthesis of ZnO NPs and UV–Vis spectroscopy profile of biosynthesized ZnO NPs

**Figure 1:**
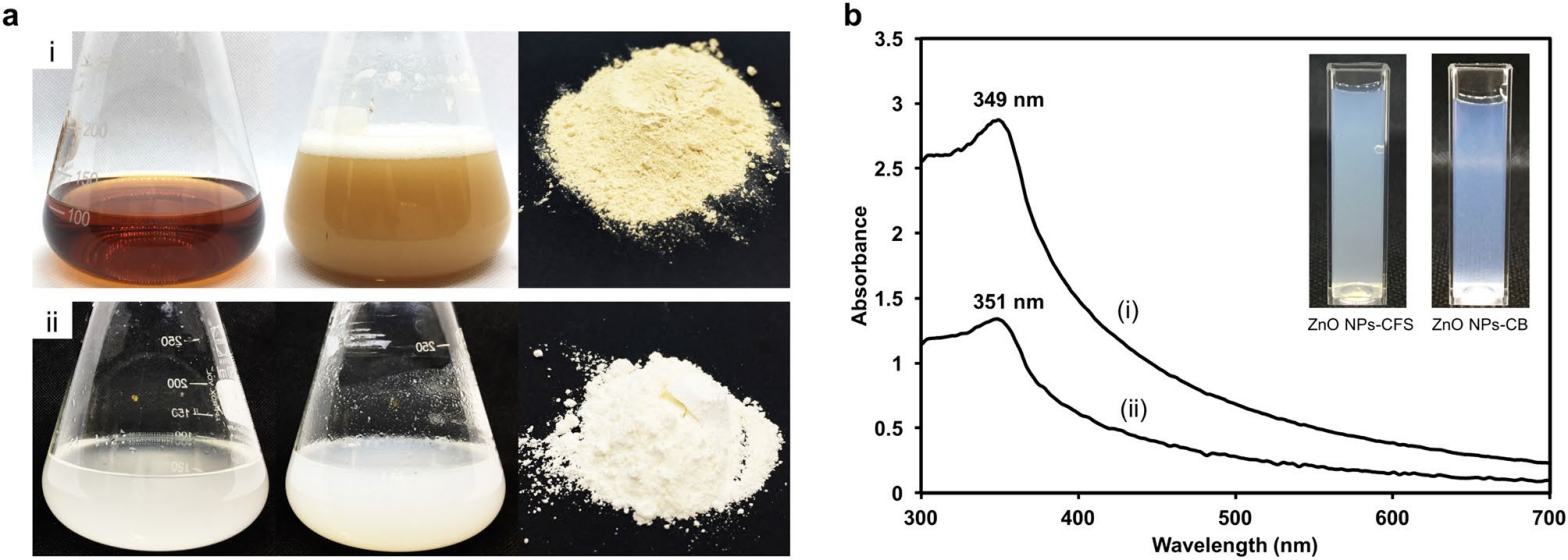
(**A**) Reduction of Zn^2+^ to ZnO NPs by (i) cell-free supernatant and (ii) cell biomass of *L. plantarum* TA4. The collected ZnO NPs were washed repeatedly with distilled water and followed by ethanol and then dried at 100 °C overnight to obtain a white powder. (**B**) UV–Vis spectrum of (**a**) ZnO NPs-CFS and (**b**) ZnO NPs-CB. The inset image shows visual observation of both biosynthesized ZnO NPs dispersed in deionized water. The images in (**A**, **B**) were taken by H.M.Y and compiled in Adobe photoshop (version CS6).

In recent years, the exploitation of microorganisms in the synthesis of NPs has received considerable attention as an alternative to the chemical and physical methods. The utilization of biological substances from the microorganism leads to the elimination of expensive and harsh chemicals. The synthesis process of NPs can be achieved using extracellular or intracellular biological compounds from microorganisms. In this study, the aqueous Zn^2+^ was reduced to ZnO NPs when added to CB and CFS derived from *L. plantarum* TA4. The preliminary confirmation for the formation of ZnO NPs-CFS was determined by visual observation of the reaction mixture of CFS and Zn^2+^. This is indicated by the appearance of white precipitates deposited at the bottom of flasks (Fig. 1a). Meanwhile, the CB-mediated synthesis was also screened by the observation of color changes in the reaction suspension. Prior to incubation, the color of CB in distilled water was whitish clear, and after the addition of Zn^2+^, the color of the reaction was whitish cloudy, implying the reduction of Zn^2+^ to ZnO NPs (Fig. 1a).

The reduction of Zn^2+^ to ZnO NPs was proposed to occur by the action of biological compounds secreted into the supernatant by the bacteria and also functional group present on the bacterial cell. Markus et al.^28^ demonstrated the involvement of protein and functional groups (carboxylate) on *L. kimchicus* DCY51, which is responsible for the reduction of gold nanoparticles. Meanwhile, Kato et al.^29^ identified Lacto-*N*-triose and lactic acid in the supernatant of *L. casei* as the reducing agent for gold nanoparticle synthesis. The current results in this study suggested that the reducing effect of proteins present in CFS and CB suspension, with the concentrations of 2.79 ± 0.11 mg/mL and 1.94 ± 0.20 mg/mL, respectively, were involved in the synthesis process of ZnO NPs. Similarly, Li et al.^30^ demonstrated the biosynthesis of gold nanoparticles using protein extract from *Deinococcus radiodurans.* They suggested that functional groups such as –NH_2_, O–H, and –COOH derived from protein act as binding sites to facilitate the gold reduction process.

After the recovery of ZnO NPs from the CFS and CB route, the obtained white powder was dispersed in dH_2_O for subsequent analysis. The reduction of Zn^2+^ to ZnO NPs was monitored by UV–Vis spectroscopy of the colloidal solution in the range of 300–700 nm. The UV–Vis spectrum of ZnO NPs-CFS and ZnO NPs-CB sample (Fig. 1b) showed a profound peak at 349 and 351 nm, respectively, which is the typical characteristic of ZnO NPs and confirmed the formation of ZnO NPs. Likewise, prior studies of ZnO NPs’ biosynthesis employing microorganisms reported the same range of absorption peaks^31,32^.

### FTIR analysis

**Figure 2:**
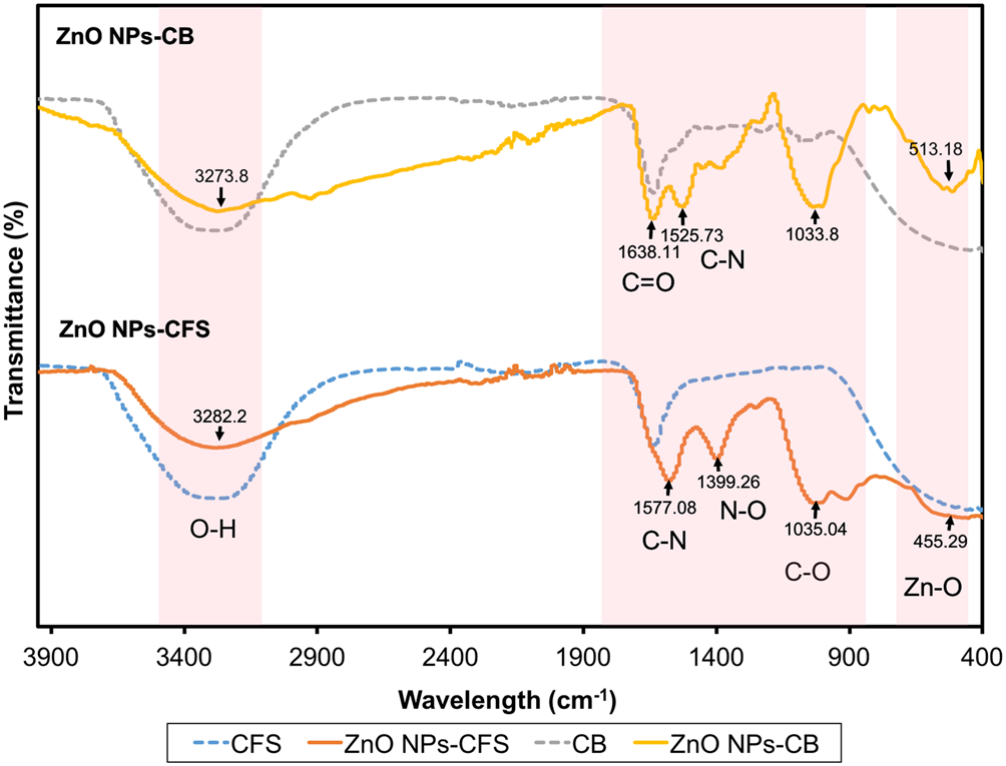
FTIR spectra of cell biomass (CB) and cell-free supernatant (CFS) of *L. plantarum* TA4 and the biosynthesized ZnO NPs.

The bioactive molecules composition of CFS and CB and their distribution on the resulting ZnO NPs were examined using FTIR spectroscopy. Figure 2 illustrates the FTIR absorption spectra. A broad absorption peak found at 3282.2 and 3273.8 cm^–1^ for ZnO NPs-CFS and ZnO NPs-CB, respectively, denote the presence of the hydroxyl functional group (O–H band). This O–H group originated from protein and carbohydrates present in CB and CFS of *L. plantarum* TA4, as shown in the graph, which is therefore responsible for the reduction and ZnO NPs synthesis. Similarly, Jalal et al.^33^ have reported the detection of the O–H functional group derived from carbohydrates present in the supernatant of *Candida glabrata*, which is responsible for the synthesis process of silver NPs. Next, the absorption peaks at 1577.08 and 1525.73 cm^–1^ for ZnO NPs-CFS and ZnO NPs-CB, respectively, correspond to amide II band (C–N stretching and N–H deformation), while the absorption peak at 1638.11 cm for ZnO NPs-CB correspond to amide I (C=O stretching)^34^. These absorption peaks provide evidence which indicates that the biosynthesized ZnO NPs have stabilized with proteins secreted in the CB and CFS solutions. Several studies have reported that proteins are responsible for stabilizing ZnO NPs by preventing their agglomeration^35–37^. For example, Raliya and Tarafdar^35^ have demonstrated the synthesis of ZnO NPs using fungi, which secretes a protein that acts as a capping agent to prevent aggregation, thus, stabilizing NPs. On the other hand, the peaks at 1035.04 and 1033.80 cm^–1^ for ZnO NPs-CFS and ZnO NPs-CB, respectively, are associated with carbohydrate C–O–C ether bond of polysaccharides^38^. Furthermore, the presence of peaks at 455.29 cm^–1^ for ZnO NPs-CFS and 545.80 and 513.18 cm^–1^ for ZnO NPs-CB represents the band of zinc oxide core^34,39^, which confirms the nature of ZnO NPs in the samples. Meanwhile, these peaks were not observed in CB and CFS samples. Nevertheless, other studies have demonstrated various FTIR spectra bands of ZnO at positions of 416.14^34^, 503.00^38^, and 618.00 cm^–1^ ^40^. In short, the obtained data have led to the conclusion that bioactive molecules (hydroxyl, amine and carboxyl from proteins) in CB and CFS were involved in the reduction and stabilization of ZnO NPs. This was inferred based on the attachment of these bioactive molecules on the resulting ZnO NPs as recorded in the FTIR spectra.

### Characterization of biosynthesized ZnO NPs

#### Particle size distribution using DLS

**Figure 3:**
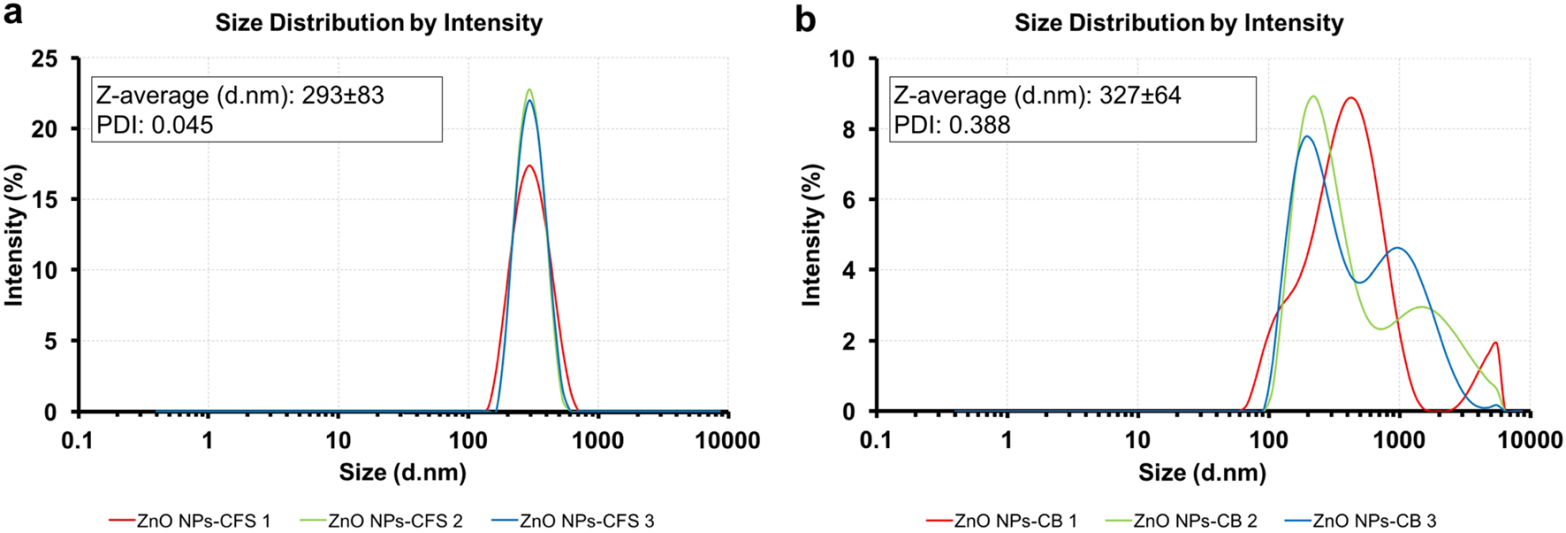
Particles size distribution of biosynthesized ZnO NPs. (**A**) ZnO NPs-CFS and (**B**) ZnO NPs-CB. The measurements were carried out in triplicate and the Z-average value obtained was generated by the software equipped with the DLS instrument.

The hydrodynamic size of biosynthesized ZnO NPs was characterized using the DLS technique (Fig. 3). The Z-average size of ZnO NPs-CFS was 292.6 ± 83.2 nm with an average PDI of 0.045 (Fig. 3a), while the Z-average size for ZnO NPs-CB was 327.4 ± 634.8 nm with a PDI of 0.388 (Fig. 3b). The DLS graph analysis of ZnO NPs-CFS revealed the presence of high, mono-dispersed NPs in the suspension that corresponded to their PDI value. In contrast, the biosynthesized ZnO NPs-CB showed various size distribution peaks, thus indicating their nature of non-uniformity in size. It should be noted that the PDI value obtained for ZnO NPs-CB was higher compared to the PDI value of ZnO NPs-CFS. A similar observation was reported by Markus et al.^28^, in which the gold nanoparticles synthesized using probiotic *L. kimchicus* DCY51 were observed to be entirely poly-dispersed with sizes varying from 40 to 300 nm. Nevertheless, the hydrodynamic size of both the biosynthesized ZnO NPs consisted of a large diameter due to the measurements taken from the metal core to the biological compound that was attached to the particle surface^28^, thus reflecting their bigger sizes.

#### HR-TEM analysis

**Figure 4:**
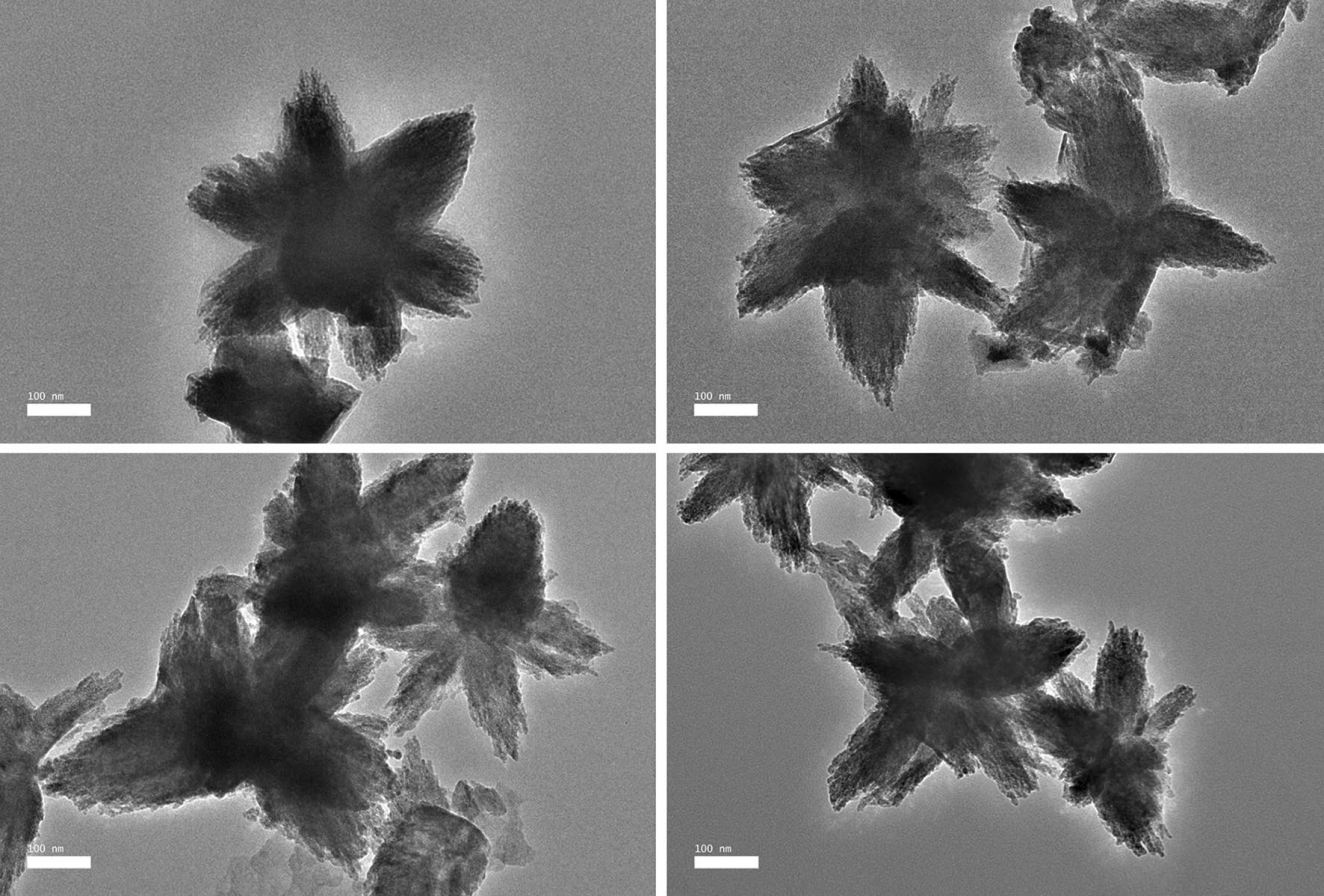
HR-TEM micrographs of biosynthesized ZnO NPs-CFS with nanoflower-shaped.

**Figure 5:**
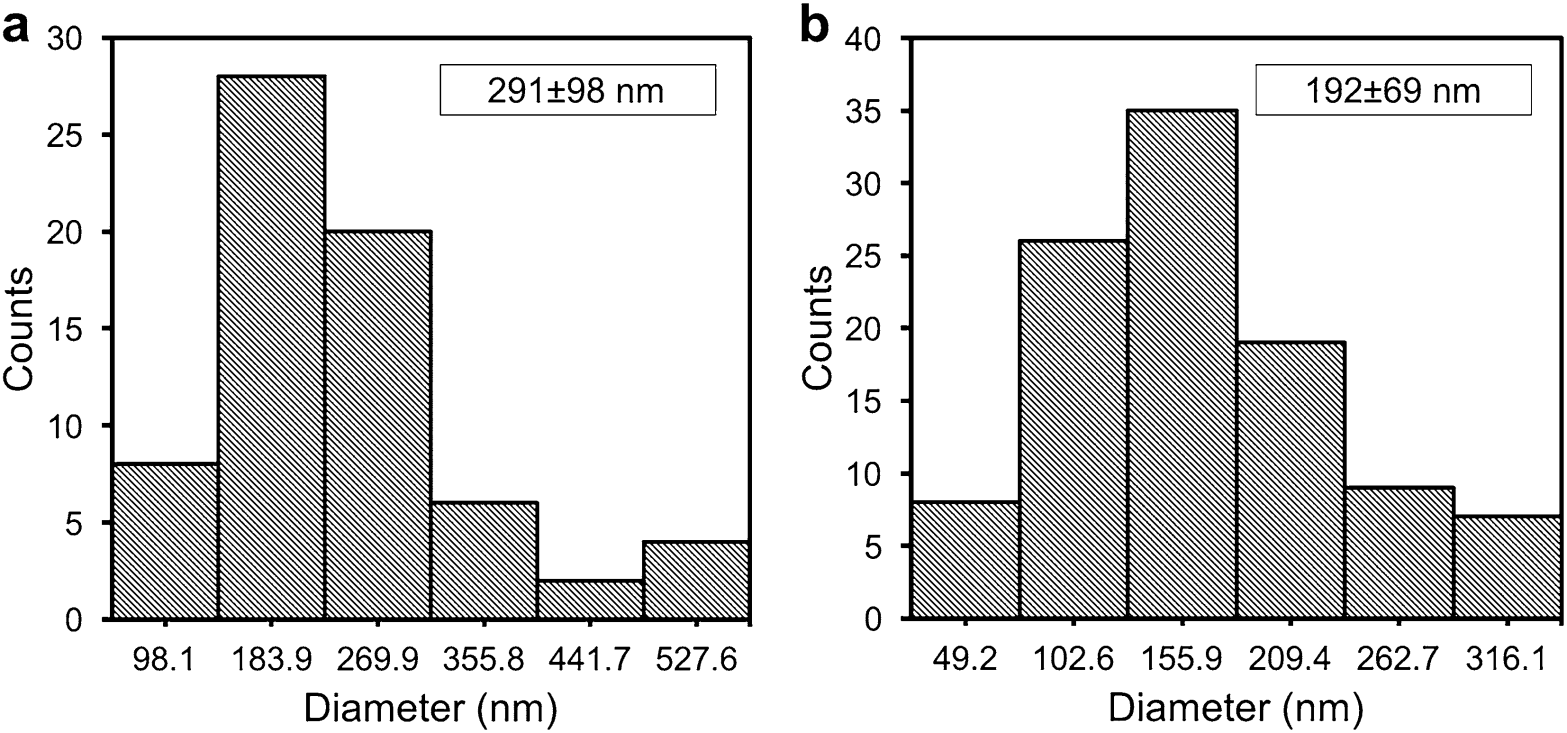
Size distribution of particles in HR-TEM micrographs of (**A**) ZnO NPs-CFS and (**B**) ZnO NPs-CB. The size distribution histogram of ZnO NPs was generated from a hundred particles (*N* = 100) for average size determinations.

The morphological shapes and sizes of the biosynthesized ZnO NPs were analyzed using HR-TEM. The HR-TEM micrographs (Fig. 4) revealed a flower pattern for ZnO NPs-CFS, with diverse sizes ranging from 152.8 to 613.5 nm and an average size of 291.1 ± 98.1 nm (Fig. 5a). The nanoflower-like ZnO NPs-CFS displayed multiple, structurally arranged petals with petal size dimensions of 83.3 ± 31.9 nm (width) and 153.8 ± 38.2 nm (length). The nanoflower-shaped ZnO NPs were also previously reported by Tripathi et al.^41^, in which an average size of 600 nm was obtained for the biosynthesized NPs using the cell biomass of *Bacillus licheniformis*. The authors also indicated that the nanoflower-shaped NPs produced in their study was as a result of the rod-like structures that agglomerated and formed a nanoflower shape. It is evident that the shape of biosynthesized NPs can be tailored by changing the reaction conditions. It was previously reported that the flower-shaped NPs could have been produced using zinc nitrate as a precursor^42^, similar to the observation in this study. Likewise, Fakhari et al.^42^ reported that the biosynthesis of ZnO NPs using zinc acetate as a precursor produced spherical NPs, while the use of zinc nitrate resulted in nanoflower-shaped NPs.

**Figure 6:**
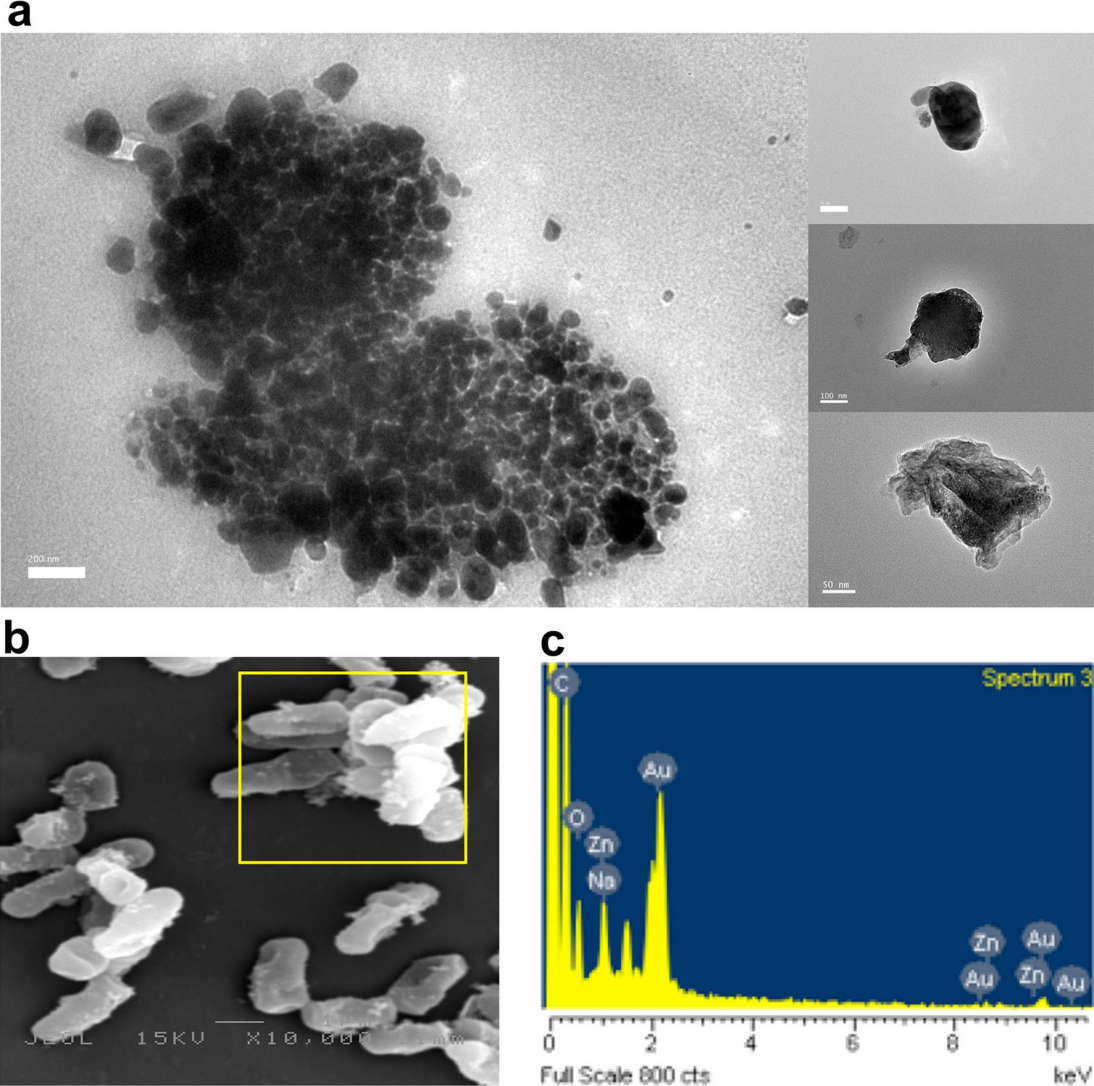
**A** HR-TEM micrograph of biosynthesized ZnO NPs-CB with various shapes. **B** SEM micrograph of *L. plantarum* TA4 exposed to Zn^2+^. **C** EDX spectra of *L. plantarum* TA4 exposed to Zn^2+^.

In contrast, the HR-TEM micrographs of ZnO NPs-CB revealed irregular-shaped NPs with a distribution of agglomerates (Fig. 6a). Moreover, FIG. 6A shows that the biosynthesized ZnO NPs were predominantly spherical, hexagonal, and oval-shaped particles, with sizes ranging from 49.2 to 369.5 nm and an average size of 191.8 ± 69.1 nm (Fig. 5b). The HR-TEM results obtained were based on the DLS analysis results that revealed the presence of poly-dispersed NPs with a variety of particle sizes (Fig. 3b), thus indicating the non-uniformity nature of NPs. Moreover, the hydrodynamic size of ZnO NPs-CB obtained using DLS was more significant than the TEM analysis due to the aggregation of NPs, as shown in the HR-TEM micrographs (Fig. 6a).

Likewise, the SEM micrograph of *L. plantarum* TA4’s cells after exposure to Zn^2+^ showed the presence of particles on the cell membrane (Fig. 6b). The formation of ZnO NPs on the bacterial cell membrane was further confirmed using the EDX analysis that revealed the presence of a zinc elemental composition peak (Fig. 6c), thus indicating the successful biosynthesis of ZnO NPs. The formation of ZnO NPs using CB was identified outside the cell, notably, on the surface of the cell membrane. Similarly, Moreno-Martin et al.^20^ reported that the formation of selenium nanoparticles was observed outside the cells of several LAB strains used in their study. It has been reported that the formation of NPs using bacterial biomass is highly dependent on the presence of functional groups on their cell surface^26,28,43^. Hence, the FTIR results obtained in this study substantiated the involvement of several functional groups present on *L. plantarum* TA4 in the synthesis process of ZnO NPs-CB. Similarly, Krol et al.^23^ also demonstrated the involvement of protein and carboxyl groups on the *L. paracasei* strain in the synthesis process of ZnO NPs.

**Figure 7:**
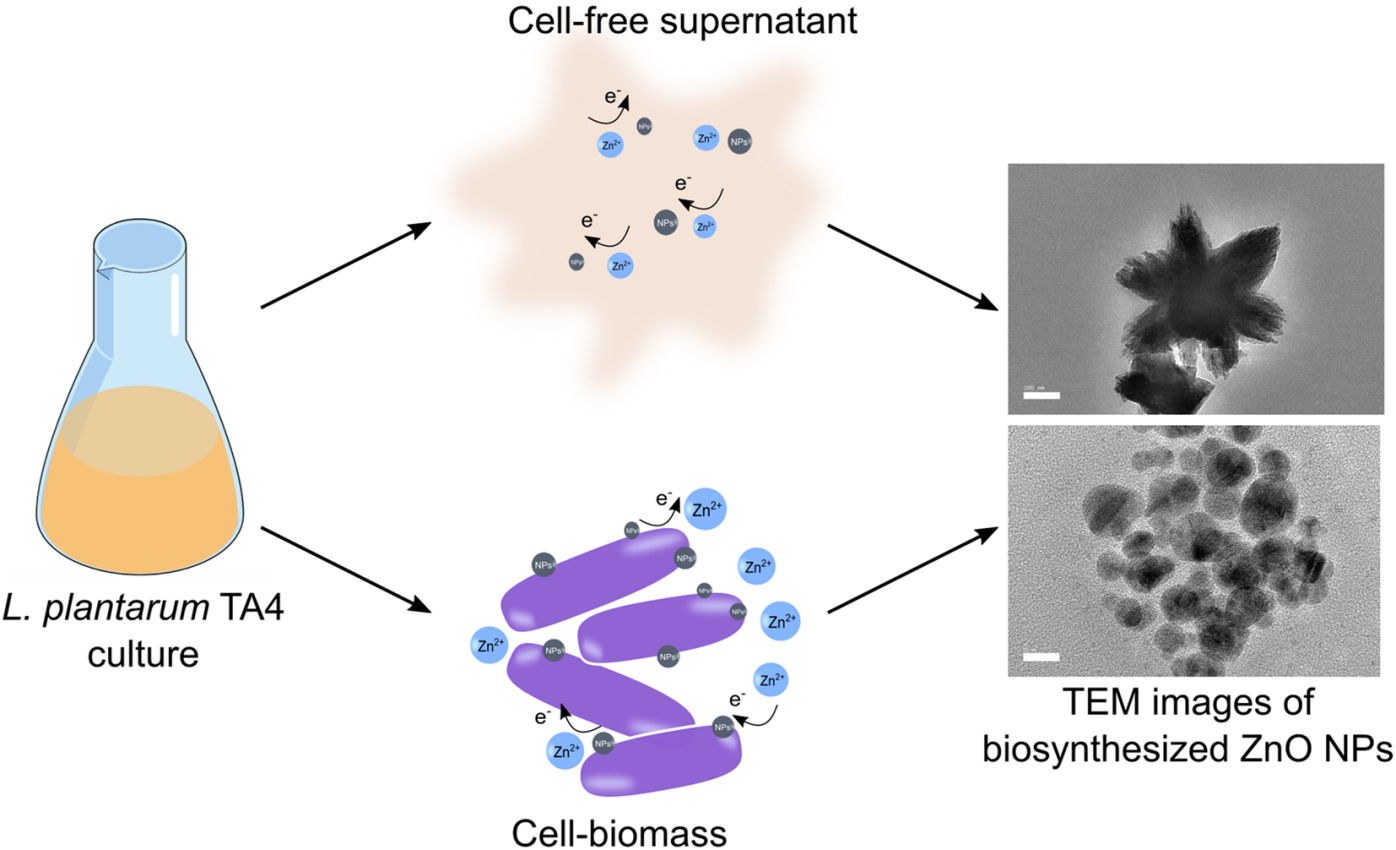
Schematic representation of biosynthesis of ZnO NPs employing cell-free supernatant (CFS) and cell biomass (CB) derived from *L. plantarum* TA4. Schematic drawings were prepared by H.M.Y using Adobe photoshop (version CS6).

In this study, the ZnO NPs sizes obtained from CFS and CB derived from *L. plantarum* TA4 were typically larger than other reported studies. The synthesis of selenium nanoparticles employing *L. acidophilus*, *L. bulgaricus*, and *L. reuteri* produced various particle size distributions of 176 ± 13 nm, 160 ± 24 nm, and 130 ± 23 nm, respectively^20^, thus indicating that the particles size distribution is dependent on the *Lactobacillus* species. Overall, the use of CFS and CB derived from *L. plantarum* TA4 resulted in the synthesis of different sizes and shapes of ZnO NPs. Figure 7 shows the schematic representation of ZnO NPs biosynthesis using CFS and CB. The CFS route produced flower-shaped ZnO NPs and this effect may be due to the presence of various biological compounds secreted in the medium such as enzymes, proteins, and organic compounds^44,45^ that act as reducing agents associated with the complex pathways involving electron transfer^46^. On the other hand, the CB route produced irregular-shaped NPs and this effect may be due to the interactions between Zn^2+^ and the functional groups present on the bacterial cell^2^ that provide a binding site for Zn^2+^ and the biosynthesis of ZnO NPs. Nevertheless, the actual biosynthetic mechanisms using both routes for the biosynthesis of ZnO NPs from *L. plantarum* TA4 require further investigation.

##### Antibacterial activity of biosynthesized ZnO NPs

**Figure 8:**
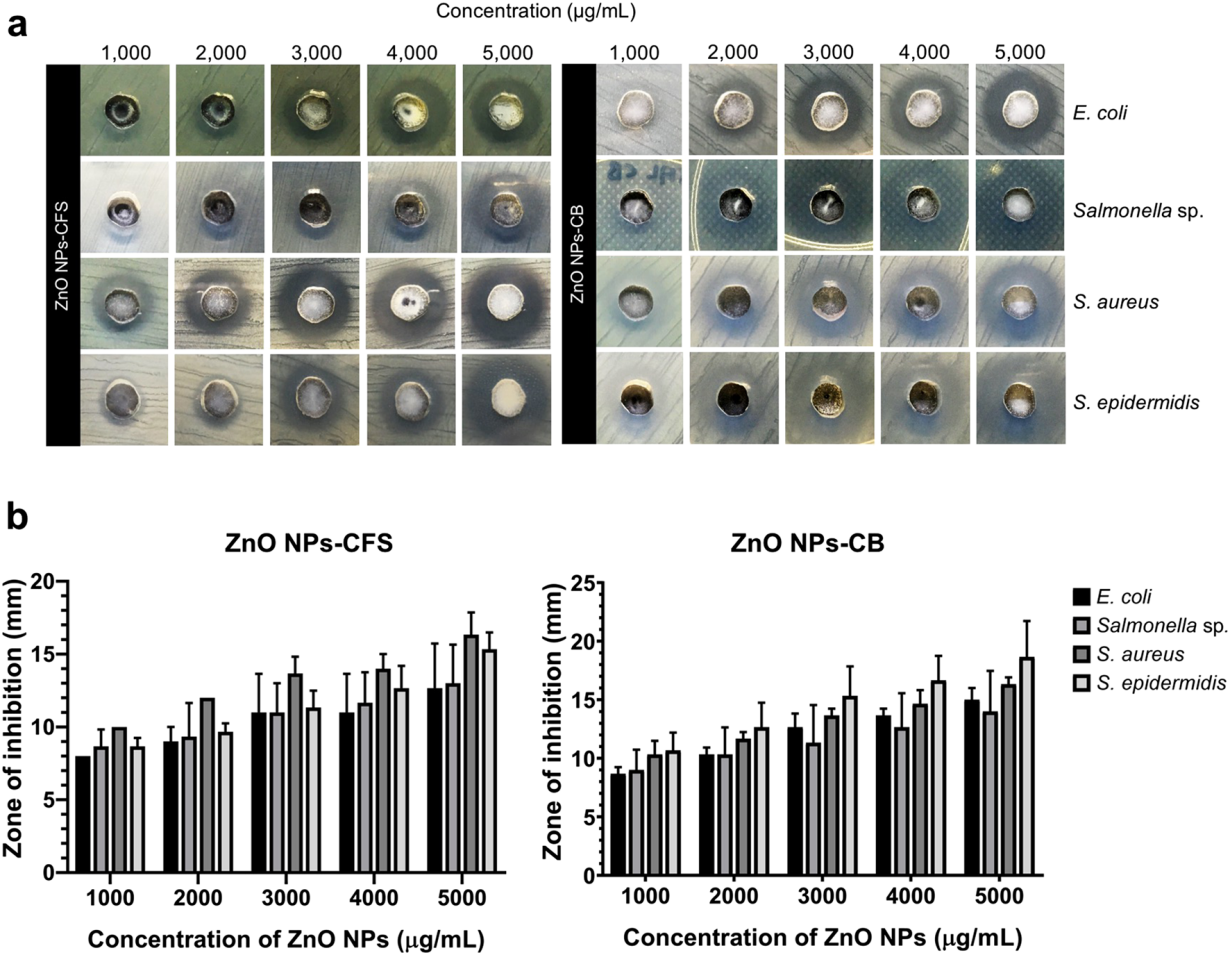
(**A**) The antibacterial effects of biosynthesized ZnO NPs evaluated by using agar well diffusion method against *E. coli*, *Salmonella* sp., *S. aureus*, and *S. epidermidis*. (**B**) Antibacterial activity of biosynthesized ZnO NPs-CFS and ZnO NPs-CB at different concentrations. The data presented as the means ± SD of three replicates.

The in vitro antibacterial efficacy of ZnO NPs has been tested in a broad spectrum of pathogenic bacteria, including *Pseudomonas aeruginosa*^47^*, Bacillus subtilis*^48^, and *Helicobacter pylori*^31^. Due to their significant antibacterial ability, ZnO NPs has been exploited in various industries, including the food industry, particularly as a feed additive and antimicrobial agent for food packaging^49^. In this study, the antibacterial activity of biosynthesized ZnO NPs-CFS and ZnO NPs-CB against Gram-negative (*E. coli* and *Salmonella* sp.) and Gram-positive (*S. aureus* and *S. epidermidis*) bacteria were carried out using agar well diffusion method (Fig. 8a). The average value of the inhibitory zone at different concentrations of ZnO NPs against the tested pathogens is presented in Fig. 8b. The biosynthesized ZnO NPs demonstrated antibacterial efficacy with varying degrees of activity. Briefly, a notable increase of inhibitory effect was observed with the increase of its concentration in both biosynthesized ZnO NPs, which was also observed in another study^39^.

Table 1 presents the MIC and MBC value of biosynthesized ZnO NPs. The results revealed that the ZnO NPs-CFS inhibits bacterial growth at ZnO NPs concentrations of 2500, 2500, 1250, and 1250 µg/mL for *E. coli*, *Salmonella sp.*, *S. aureus*, and *S. epidermidis*, respectively. Whereas, the ZnO NPs-CB inhibits bacterial growth at 625, 1250, 312.5, and 1250 µg/mL for *E. coli*, *Salmonella *sp*.*, *S. aureus*, and *S. epidermidis*, respectively. Meanwhile, the bactericidal effects of both ZnO NPs against the same tested pathogens were 8000, 4000, 2500, and 2500 µg/mL and 8000, 8000, 2500, and 2500 µg/mL for ZnO NPs-CFS and ZnO NPs-CB, respectively. Based on the results, it was found that the biosynthesized ZnO NPs were more effective against Gram-positive bacteria compared to Gram-negative bacteria. This was due to the different cell wall structures of the bacteria, where Gram-negative bacteria have a thick outer cell membrane layer that makes them resistant to ZnO NPs compared to Gram-positive bacteria^3^. Moreover, it was also suggested that the presence of lipopolysaccharide on the cell wall of Gram-negative bacteria exerted strong aversion towards the NPs, making them resistant to NPs^50^. A similar trend was observed in the study by Umar et al.^40^, where the antibacterial activity of biosynthesized ZnO NPs was reported to be more effective on *S. aureus* than on *E. coli*.

**Table 1:** Minimum inhibitory concentration (MIC) and minimum bactericidal concentration (MBC) of biosynthesized ZnO NPs against Gram-positive and Gram-negative pathogens.

NPs	Pathogen strains	MIC (µg/mL)	MBC (µg/mL)
ZnO NPs-CFS	*E. coli*	2500	8000
	*Salmonella* sp*.*	2500	4000
	*S. aureus*	1250	2500
	*S. epidermidis*	1250	2500
ZnO NPs-CB	*E. coli*	625	8000
	*Salmonella* sp*.*	1250	8000
	*S. aureus*	312.5	2500
	*S. epidermidis*	1250	2500

The antibacterial mechanisms of ZnO NPs are not fully understood, but studies suggested that the antibacterial actions were initiated by the physical contact between bacteria and metal NPs. Several mechanisms have been proposed for the antibacterial activity of ZnO NPs^2,3^, and the most suggested mechanisms are due to the formation of reactive oxygen species (ROS) and the release of Zn^2+^, which resulted in cell damage of the bacteria and cell death^3^. Besides, the cation properties of ZnO NPs enable them to attach to the negatively-charged bacterial cell surface through electrostatic interaction and subsequently damages the bacterial surface^12^. However, this action is ultimately dependent on the size of the NPs. The smaller NPs have a greater surface area and exhibit higher reactivity; thus, increasing their antibacterial efficacy compared to the larger NPs^3^. Khatami et al.^51^ reported that the biosynthesized ZnO NPs with a size of 2.8 nm demonstrated higher inhibitory activity with lower MIC value at 2.0 µg/mL against *S. aureus* and *E. coli.* Also, Abu Hanif et al.^52^ demonstrated that the synthesized ZnO NPs inhibits the growth of *E. coli* at a comparatively lower concentration of 50 µg/mL, and they suggested that the antibacterial activity of ZnO NPs is size-dependent. Contradictory to our findings, the biosynthesized ZnO NPs produced in this present study were significant compared to other studies, which resulted in bigger values for MIC and MBC.

##### Biocompatibility of biosynthesized ZnO NPs

**Figure 9:**
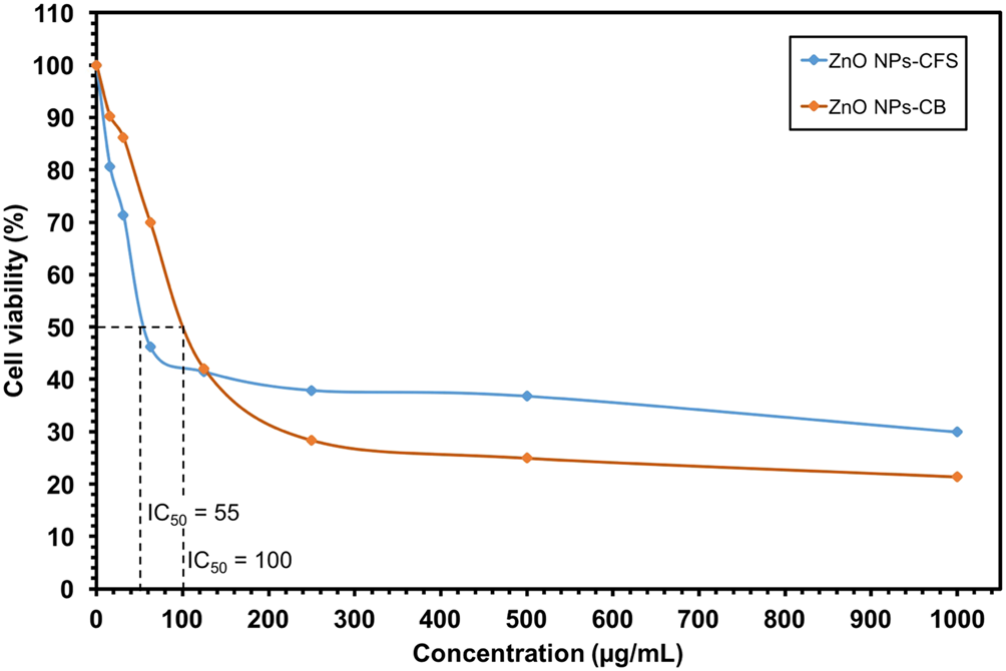
Cell viability (%) of Vero cells treated with the indicated amount of ZnO NPs and measured after 24 h of the exposure period.

**Figure 10:**
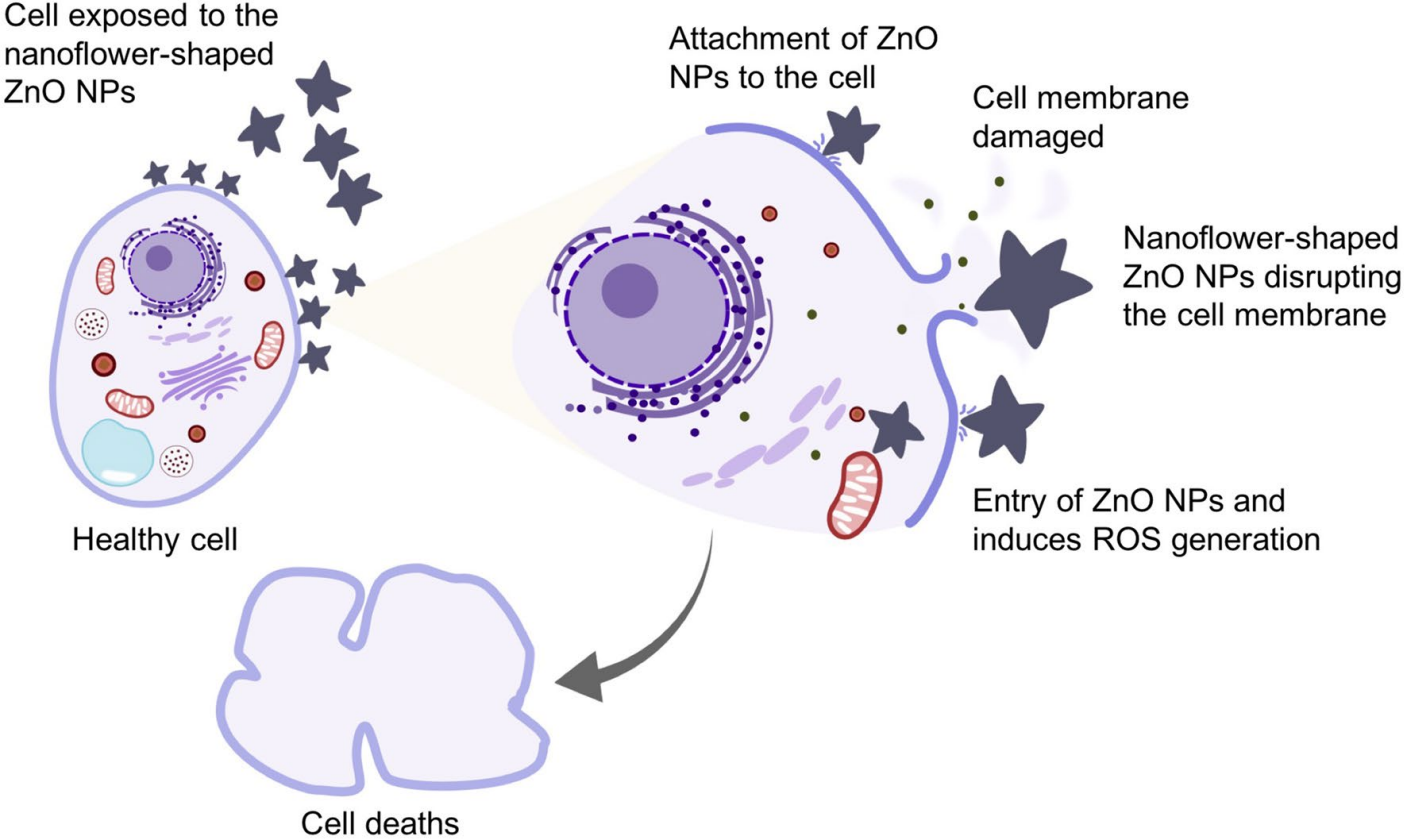
Schematic representation of the cytotoxic action of nanoflower-shaped ZnO NPs. The direct interaction between cell and ZnO NPs damages the cell membrane integrity, resulting in the cell rupture and leakage of intracellular contents. Eventually, the ZnO NPs penetrates the cell and damages the cellular components by inducing the reactive oxygen species (ROS) resulting in cells death. Schematic drawings were prepared by H.M.Y using Adobe photoshop (version CS6).

ZnO NPs are a multifunctional element that is widely used in many applications, such as feed supplements in the animal industry^12,53^. However, its biocompatibility with biological organisms is a major concern. ZnO NPs have been reported to exert cytotoxic effects on various cell lines^17,31,37^. Nevertheless, the cytotoxic effects are highly dependent on many parameters, including the characteristics of NPs (shape and size), synthesis methods, and the type of cell line used^2,54^. Consequently, most of the studies have focused on reducing their toxic effects and improving its functionality. In this study, the MTT assay was utilized to investigate the cytotoxic effects of the biosynthesized ZnO NPs-CFS and ZnO NPs-CB on the Vero cell line. Based on the results in Fig. 9, ZnO NPs-CFS and ZnO NPs-CB exhibited cytotoxic effects against the Vero cell line in a dose-dependent manner. The cell viability decreased significantly at the concentrations of 62.5, 125, 250, 500, and 1000 µg/mL (46.17%, 41.45%, 37.90%, 36.79%, and 29.94%, respectively) when treated with ZnO NPs-CFS. A similar pattern was observed for ZnO NPs-CB, whereby the cell viability decreased significantly at the concentrations of 125, 250, 500, and 1000 µg/mL (41.99%, 28.34%, 24.94%, and 21.36%, respectively). The cytotoxicity was recorded as the NPs concentration that causes 50% growth inhibition (IC_50_) of the cell line. The IC_50_ value of ZnO NPs-CFS and ZnO NPs-CB on Vero cells after 24 h exposure occurs at 55.0 and 100.0 µg/mL, respectively. A distinct cytotoxic effect was exhibited by the ZnO NPs-CFS, which might be due to the nanoflower-shape of the NPs. It has been reported that the cytotoxicity of NPs is affected by its shape^55–58^ besides the size and surface charge. The sharp nanostructures of the flower-shaped NPs exhibited a strong ability to damage the cell membrane, which subsequently causes cell death. Figure 10 presents the possible cytotoxicity mechanisms of the nanoflower-shaped NPs on cell. Paino et al.^59^ reported that the nanoflower-shaped of ZnO NPs demonstrated a pronounced toxic effect on HeLa cancer cells at a lower concentration of 10.0 µg/mL. Likewise, the flower-shaped biosynthesized ZnO NPs showed cytotoxic effects towards cancerous human lung alveolar epithelial cell line A549 and human lung alveolar epithelial cell line MRC-5 cell line at the concentrations of 50 and 100 µg/mL, respectively^34^. Therefore, our results suggested that the cytotoxicity of NPs was shape-dependent, following other studies^55,56,58,59^. Besides, the main cytotoxicity mechanisms of the metal NPs were thought to be associated with oxidative stress-mediated DNA damage and lipid peroxidation that causes cell apoptosis^60^ and the release of Zn^2+^^61^.

On the other hand, higher cell viability was observed for both the biosynthesized ZnO NPs at concentrations ranging from 15.63 to 62.5 µg/mL; thus, indicating their biocompatibility characteristics. These findings were consistent with the results by Ebadi et al.^34^, whereby the biosynthesized ZnO NPs using the cell extract of cyanobacterium *Nostoc* sp. EA03 showed a lesser cytotoxic effect on the proliferation of A549 and MRC-5 cell lines at lower concentrations (10, 25, and 50 µg/mL). In contrast, Abbasi et al.^62^ showed that the biosynthesized ZnO NPs displayed a maximum cytotoxic effect on hepatocellular carcinoma (HepG2) human cells at a lower concentration of 100 µg/mL. Nevertheless, our results revealed that the IC_50_ value was more than 50 µg/mL compared to those chemically synthesized ZnO NPs^34,63,64^; hence, considered as biocompatible to the cell line.

The low cytotoxic effects of biosynthesized ZnO NPs have been previously reported^31^. Markus et al.^28^ demonstrated a lower cytotoxic activity of LAB-mediated synthesis of gold nanoparticles on murine macrophage (RAW264.7) and human colon cancer cell lines (HT29) after 72 h of treatment. The authors suggested that the lower cytotoxic effects were associated with the biomolecules from *L. kimchicus* DCY51 that coated the NPs; thus, reducing their toxic effects. Moreover, Darvishi et al.^65^ compared the cytotoxic effects between the green (walnut extract) and chemically synthesized ZnO NPs on human skin fibroblasts. The results showed that the green synthesized ZnO NPs were less toxic to the cells as compared to the chemically synthesized ZnO NPs. The authors also indicated that the biological compounds from the walnut extract acted as a capping agent that lowered the toxicity. Similarly, the silver nanoparticles synthesized by *Penicillium aculeatum* Su1 demonstrated higher biocompatibility with normal human cells (HBE cells) compared to the silver ions that showed high cytotoxic effects even at lower doses^66^. Based on these studies, it is postulated that biological molecules can act as a layer that lowers the cytotoxic activity, as evidenced in this study. Therefore, the biocompatibility results of biosynthesized ZnO NPs obtained in this study signify their essential role in biological applications.

## Conclusions

In conclusion, an environmentally friendly and cheaper method was unveiled for the biosynthesis of ZnO NPs using zinc-tolerant probiotic *L. plantarum* TA4 derived from CFS and CB. The formation of ZnO NPs was confirmed by the presence of a peak in the UV–Vis spectrum. The presence of hydroxyl, amine, and carboxyl groups from proteins derived from CFS and CB that were detected on the surface of ZnO NPs using FTIR played a significant role in the reduction process. DLS analysis on both biosynthesized ZnO NPs showed mono-dispersed NPs and HR-TEM micrographs that revealed flower- and irregular-shaped patterns for ZnO NPs-CFS and ZnO NPs-CB, respectively. The antibacterial activity against Gram-positive and Gram-negative pathogens also revealed that the inhibitory and bactericidal efficacy of both biosynthesized ZnO NPs were concentration-dependent. The biosynthesized ZnO NPs-CFS and ZnO NPs-CB demonstrated biocompatibility in the MTT assay using Vero cells with higher cell viability observed after 24 h at the concentration ranging from 15.63 to 62.5 µg/mL. In this present study, the application of CFS from *L. plantarum* TA4 was found convenient in terms of NPs purification in comparison with CB-derived ZnO NPs. CB-derived ZnO NPs required multiple steps, including several centrifugations and ultrasonication cycles for NPs recovery, making the purification step complicated. It was recommended to use CFS of *L. plantarum* TA4 for the synthesis process of ZnO NPs for future applications.

## References

[1] Jiang J, Pi J, Cai J (2018). The advancing of zinc oxide nanoparticles for biomedical applications. Bioinorg. Chem. Appl..

[2] Mohd Yusof H, Mohamad R, Zaidan UH, Abdul Rahman N (2019). Microbial synthesis of zinc oxide nanoparticles and their potential application as an antimicrobial agent and a feed supplement in animal industry : a review. J. Anim. Sci. Biotechnol..

[3] Sirelkhatim A (2015). Review on zinc oxide nanoparticles: antibacterial activity and toxicity mechanism. Nano-Micro Lett..

[4] Huang Y, Mei L, Chen X, Wang Q (2018). Recent developments in food packaging based on nanomaterials. Nanomaterials.

[5] Sun Q, Li J, Le T (2018). Zinc oxide nanoparticle as a novel class of antifungal agents: current advances and future perspectives. J. Agric. Food Chem..

[6] Seil JT, Webster TJ (2012). Antimicrobial applications of nanotechnology: methods and literature. Int. J. Nanomed..

[7] Wang C (2017). Zinc oxide nanoparticles as a substitute for zinc oxide or colistin sulfate: effects on growth, serum enzymes, zinc deposition, intestinal morphology and epithelial barrier in weaned piglets. PLoS ONE.

[8] Zhao C (2014). Effects of dietary zinc oxide nanoparticles on growth performance and antioxidative status in broilers. Biol. Trace Elem. Res.

[9] Abedini M, Shariatmadari F, Karimi Torshizi MA, Ahmadi H (2018). Effects of zinc oxide nanoparticles on the egg quality, immune response, zinc retention, and blood parameters of laying hens in the late phase of production. J. Anim. Physiol. Anim. Nutr. (Berl).

[10] Abedini M, Shariatmadari F, Torshizi MAK, Ahmadi H (2017). Effects of a dietary supplementation with zinc oxide nanoparticles, compared to zinc oxide and zinc methionine, on performance, egg quality, and zinc status of laying hens. Livest. Sci..

[11] Khajeh Bami M, Afsharmanesh M, Salarmoini M, Tavakoli H (2018). Effect of zinc oxide nanoparticles and *Bacillus coagulans* as probiotic on growth, histomorphology of intestine, and immune parameters in broiler chickens. Comp. Clin. Path..

[12] Mohd Yusof H, Mohamad R, Zaidan UH, Abdul Rahman NA (2019). Microbial synthesis of zinc oxide nanoparticles and their potential application as an antimicrobial agent and a feed supplement in animal industry: a review. J. Anim. Sci. Biotechnol..

[13] Liu J (2017). Zinc oxide nanoparticles induce toxic responses in human neuroblastoma SHSY5Y cells in a size-dependent manner. Int. J. Nanomed..

[14] Fouda A, EL-Din-Hassan S, Salem SS, Shaheen TI (2018). In-vitro cytotoxicity, antibacterial, and UV protection properties of the biosynthesized Zinc oxide nanoparticles for medical textile applications. Microb. Pathog..

[15] Ng CT (2017). Zinc oxide nanoparticles exhibit cytotoxicity and genotoxicity through oxidative stress responses in human lung fibroblasts and *Drosophila melanogaster*. Int. J. Nanomed..

[16] Burns AA (2009). Fluorescent silica nanoparticles with efficient urinary excretion for nanomedicine. Nano Lett..

[17] Wahab R (2016). Self-styled ZnO nanostructures promotes the cancer cell damage and supresses the epithelial phenotype of glioblastoma. Sci. Rep..

[18] Agarwal H, Menon S, Venkat Kumar S, Rajeshkumar S (2018). Mechanistic study on antibacterial action of zinc oxide nanoparticles synthesized using green route. Chem. Biol. Interact..

[19] Kitching M, Ramani M, Marsili E (2015). Fungal biosynthesis of gold nanoparticles: mechanism and scale up. Microb. Biotechnol..

[20] Moreno-Martin G, Pescuma M, Pérez-Corona T, Mozzi F, Madrid Y (2017). Determination of size and mass-and number-based concentration of biogenic SeNPs synthesized by lactic acid bacteria by using a multimethod approach. Anal. Chim. Acta.

[21] Garmasheva I (2016). *Lactobacillus* species mediated synthesis of silver nanoparticles and their antibacterial activity against opportunistic pathogens in vitro. BioImpacts.

[22] Chapot-Chartier M-P, Kulakauskas S (2014). Cell wall structure and function in lactic acid bacteria. Microb. Cell Fact..

[23] Król A, Railean-Plugaru V, Pomastowski P, Złoch M, Buszewski B (2018). Mechanism study of intracellular zinc oxide nanocomposites formation. Colloids Surf. A Physicochem. Eng. Asp..

[24] Selvarajan E, Mohanasrinivasan V (2013). Biosynthesis and characterization of ZnO nanoparticles using *Lactobacillus plantarum* VITES07. Mater. Lett..

[25] Mohd Yusof H, Mohamad R, Zaidan UH, Abdul Rahman N (2020). Sustainable microbial cell nanofactory for zinc oxide nanoparticles production by zinc-tolerant probiotic *Lactobacillus plantarum* strain TA4. Microb. Cell Fact..

[26] Liang X (2019). Fungal formation of selenium and tellurium nanoparticles. Appl. Microbiol. Biotechnol..

[27] Rajeswaran S, Somasundaram Thirugnanasambandan S, Rengasamy Subramaniyan S, Kandasamy S, Vilwanathan R (2019). Synthesis of eco-friendly facile nano-sized zinc oxide particles using aqueous extract of *Cymodocea serrulata* and its potential biological applications. Appl. Phys. A.

[28] Markus J (2016). Intracellular synthesis of gold nanoparticles with antioxidant activity by probiotic *Lactobacillus kimchicus* DCY51 isolated from Korean kimchi. Enzyme Microb. Technol..

[29] Kato Y, Yoshimura E, Suzuki M (2019). Synthesis of gold nanoparticles by extracellular components of *Lactobacillus casei*. Chem. Select.

[30] Li J (2016). Biosynthesis of gold nanoparticles by the extreme bacterium *Deinococcus radiodurans* and an evaluation of their antibacterial properties. Int. J. Nanomed..

[31] Saravanan M (2018). Green synthesis of anisotropic zinc oxide nanoparticles with antibacterial and cytofriendly properties. Microb. Pathog..

[32] Ezealisiji KM, Siwe-Noundou X, Maduelosi B, Nwachukwu N, Krause RWM (2019). Green synthesis of zinc oxide nanoparticles using *Solanum torvum* (L.) leaf extract and evaluation of the toxicological profile of the ZnO nanoparticles–hydrogel composite in Wistar albino rats. Int. Nano Lett..

[33] Jalal M (2018). Biosynthesis of silver nanoparticles from *Oropharyngeal Candida glabrata* isolates and their antimicrobial activity against clinical strains of bacteria and fungi. Nanomaterials.

[34] Ebadi M (2019). A bio-inspired strategy for the synthesis of zinc oxide nanoparticles (ZnO NPs) using the cell extract of *Cyanobacterium Nostoc* sp. EA03: from biological function to toxicity evaluation. RSC Adv..

[35] Raliya R, Tarafdar JC (2013). ZnO nanoparticle biosynthesis and its effect on phosphorous-mobilizing enzyme secretion and gum contents in Clusterbean (*Cyamopsis tetragonoloba* L.). Agric. Res..

[36] Sarkar J, Ghosh M, Mukherjee A, Chattopadhyay D, Acharya K (2014). Biosynthesis and safety evaluation of ZnO nanoparticles. Bioprocess. Biosyst. Eng..

[37] Balraj B (2017). Synthesis and characterization of Zinc Oxide nanoparticles using marine *Streptomyces* sp. with its investigations on anticancer and antibacterial activity. Res. Chem. Intermed..

[38] Khalafi T, Buazar F, Ghanemi K (2019). Phycosynthesis and enhanced photocatalytic activity of zinc oxide nanoparticles toward organosulfur pollutants. Sci. Rep..

[39] Ogunyemi SO (2019). Green synthesis of zinc oxide nanoparticles using different plant extracts and their antibacterial activity against *Xanthomonas oryzae* pv. oryzae. Artif. Cells Nanomed. Biotechnol..

[40] Umar H, Kavaz D, Rizaner N (2018). Biosynthesis of zinc oxide nanoparticles using *Albizia lebbeck* stem bark, and evaluation of its antimicrobial, antioxidant, and cytotoxic activities on human breast cancer cell lines. Int. J. Nanomed..

[41] Tripathi RM (2014). ZnO nanoflowers: novel biogenic synthesis and enhanced photocatalytic activity. J. Photochem. Photobiol. B Biol..

[42] Fakhari S, Jamzad M, Kabiri Fard H (2019). Green synthesis of zinc oxide nanoparticles: a comparison. Green Chem. Lett. Rev..

[43] Zonaro E (2017). *Ochrobactrum* sp. MPV1 from a dump of roasted pyrites can be exploited as bacterial catalyst for the biogenesis of selenium and tellurium nanoparticles. Microb. Cell Fact..

[44] Fang X, Wang Y, Wang Z, Jiang Z, Dong M (2019). Microorganism assisted synthesized nanoparticles for catalytic applications. Energies.

[45] Guilger-Casagrande M, de Lima R (2019). Synthesis of silver nanoparticles mediated by fungi: a review. Front. Bioeng. Biotechnol..

[46] Gudikandula K, Vadapally P, Singara Charya MA (2017). Biogenic synthesis of silver nanoparticles from white rot fungi: their characterization and antibacterial studies. OpenNano.

[47] Jayaseelan C (2012). Novel microbial route to synthesize ZnO nanoparticles using *Aeromonas hydrophila* and their activity against pathogenic bacteria and fungi. Spectrochim. Acta Part A Mol. Biomol. Spectrosc..

[48] Jiang W, Mashayekhi H, Xing B (2009). Bacterial toxicity comparison between nano- and micro-scaled oxide particles. Environ. Pollut..

[49] Espitia PJP (2012). Zinc oxide nanoparticles: synthesis, antimicrobial activity and food packaging applications. Food Bioprocess. Technol..

[50] Gunti L, Dass RS, Kalagatur NK (2019). Phytofabrication of selenium nanoparticles from *Emblica officinalis* fruit extract and exploring its biopotential applications: antioxidant, antimicrobial, and biocompatibility. Front. Microbiol..

[51] Khatami M, Alijani HQ, Heli H, Sharifi I (2018). Rectangular shaped zinc oxide nanoparticles: green synthesis by Stevia and its biomedical efficiency. Ceram. Int..

[52] Hanif M (2019). Enhanced photocatalytic and antibacterial performance of ZnO nanoparticles prepared by an efficient thermolysis method. Catalysts.

[53] Swain PS, Rao SBN, Rajendran D, Dominic G, Selvaraju S (2016). Nano zinc, an alternative to conventional zinc as animal feed supplement: a review. Anim. Nutr..

[54] Dincă V (2020). Biocompatible pure ZnO nanoparticles-3D bacterial cellulose biointerfaces with antibacterial properties. Arab. J. Chem..

[55] Steckiewicz KP (2019). Impact of gold nanoparticles shape on their cytotoxicity against human osteoblast and osteosarcoma in in vitro model. Evaluation of the safety of use and anti-cancer potential. J. Mater. Sci. Mater. Med..

[56] Lee YJ, Ahn E-Y, Park Y (2019). Shape-dependent cytotoxicity and cellular uptake of gold nanoparticles synthesized using green tea extract. Nanoscale Res. Lett..

[57] Woźniak A (2017). Size and shape-dependent cytotoxicity profile of gold nanoparticles for biomedical applications. J. Mater. Sci. Mater. Med..

[58] Enea M (2019). A multiparametric study of gold nanoparticles cytotoxicity, internalization and permeability using an in vitro model of blood–brain barrier. Influence of size, shape and capping agent. Nanotoxicology.

[59] Paino IMM, Gonçalves FJ, Souza FL, Zucolotto V (2016). Zinc oxide flower-like nanostructures that exhibit enhanced toxicology effects in cancer cells. ACS Appl. Mater. Interfaces.

[60] Król A, Pomastowski P, Rafińska K, Railean-Plugaru V, Buszewski B (2017). Zinc oxide nanoparticles: synthesis, antiseptic activity and toxicity mechanism. Adv. Coll. Interface. Sci..

[61] Ferrone E, Araneo R, Notargiacomo A, Pea M, Rinaldi A (2019). ZnO nanostructures and electrospun ZnO–polymeric hybrid nanomaterials in biomedical, health, and sustainability applications. Nanomaterials.

[62] Abbasi BH (2019). Green bio-assisted synthesis, characterization and biological evaluation of biocompatible ZnO NPs synthesized from different tissues of milk thistle (*Silybum marianum*). Nanomaterials.

[63] Díaz de León CL (2017). Synthesis by sol–gel and cytotoxicity of zinc oxide nanoparticles using wasted alkaline batteries. Dig. J. Nanomater. Biostruct..

[64] Alavi SJ, Khalili N, Kazemi Oskuee R, Verma KD, Darroudi M (2015). Role of polyethyleneimine (PEI) in synthesis of zinc oxide nanoparticles and their cytotoxicity effects. Ceram. Int..

[65] Darvishi E, Kahrizi D, Arkan E (2019). Comparison of different properties of zinc oxide nanoparticles synthesized by the green (using *Juglans regia* L. leaf extract) and chemical methods. J. Mol. Liq..

[66] Ma L (2017). Optimization for extracellular biosynthesis of silver nanoparticles by *Penicillium aculeatum* Su1 and their antimicrobial activity and cytotoxic effect compared with silver ions. Mater. Sci. Eng. C.

